# Integrated transcriptomic and metabolomic analyses reveal the mechanism by which quercetin inhibits reflux esophagitis in rats

**DOI:** 10.1371/journal.pone.0321959

**Published:** 2025-05-06

**Authors:** Zheng Guo, Yanping Tang, Mingli Li, Lei Yang, Lei Liu, Peicai Li, Siyu Liu

**Affiliations:** 1 Graduate School, Tianjin University of Traditional Chinese Medicine, Tianjin, China; 2 Department of Gastroenterology, Tianjin Nankai Hospital, Tianjin, China; 3 Graduate School, Tianjin Medical University, Tianjin, China; South China Agricultural University, CHINA

## Abstract

Quercetin relieved symptoms in rats with reflux esophagitis (RE), but the underlying mechanism remains unclear. Quercetin attenuated esophageal mucosal inflammation in RE rats by inhibiting the production of the inflammatory factors interleukin-1*β* (IL-1*β*) and interleukin-6 (IL-6). Additionally, through transcriptomic and metabolomic analysis, we found that metabolites related to bile acid metabolism, such as taurine, taurocholic acid, and nicotinamide, were closely associated with RE in rats. Quercetin reduced the expression of bile acid-related genes such as *Cd38*, seizure related 6 homolog like 2 (*Sez6l2*), and nitric oxide synthase 2 (*Nos2*), which may be characteristic genes and therapeutic targets for RE.

## Introduction

Reflux esophagitis (RE) is a gastrointestinal disorder characterized by the reflux of gastric or duodenal contents into the esophagus, resulting in pathological changes, including inflammation and erosion of the esophageal mucosa [1]. The pathogenesis of RE is complex and involves multiple processes, such as bile acid reflux, oxidative stress, and inflammatory responses [2]. Proton pump inhibitors (PPIs), which block the secretion of acid in the stomach, are the mainstay of treatment for RE. However, patients who take PPIs for long periods only get partial relief from their symptoms and are prone to relapse [3]. In addition, clinical studies have shown that RE patients often encounter mood disorders, such as anxiety and depression, which negatively affect their treatment outcomes and quality of life [4,5]. Traditional Chinese medicine can improve esophageal mucosal inflammation and regulate emotions in patients with RE, compensating for the deficiencies of pure western medical treatment [6].

RE is a type of gastroesophageal reflux disease (GERD). Treatment includes medical, endoscopic, and surgical therapy. PPIs remain the preferred first-line agents. Adjunctive medications include alginate antacids for symptoms, H2-receptor antagonists for symptoms at night, baclofen for reflux, and antiarrhythmic drugs for paroxysmal symptoms. And neuromodulating drugs to treat reflux patients with reflux hypersensitivity. Endoscopy should be performed if the response to PPIs is poor, or transoral incisional fundoplication is an effective endoscopic option in carefully selected patients, or laparoscopic fundoplication with magnetic sphincter augmentation is an effective surgical option [7]. However, PPIs do not inhibit bile reflux. In a randomized trial of patients with refractory gastro-esophageal reflux, adding 1500mg of the bile acid sequestrant IW-3718 to labeled doses of PPIs resulted in significantly fewer heartburn symptoms than adding placebo, and also reduced reflux symptoms [8]. We may explore bile acid reflux drugs to treat RE.

Quercetin, a ubiquitous flavonoid antioxidant with various pharmacological effects that include anti-inflammatory and antioxidant activities, has been discovered in diverse traditional Chinese medicines such as *Cuscuta reflexa* and *Psoralea corylifolia* [9,10]. Quercetin inhibited the expression of several inflammatory factors, including nuclear factor-kappa B (NF-κB) and interleukin-6 (IL-6), thereby alleviating the inflammatory response of the esophageal mucosa [11].

Recently, rapid advances in omics technologies have made transcriptomics and metabolomics robust tools for investigating pathophysiological processes in RE and exploring potential therapeutic targets. Transcriptomic and proteomic analyses identified cytokine-induced neutrophil chemoattractant 1–3 (CINC1–3) and interleukin 1*β* (IL-1*β*) as major mediators of GERD [12]. Microarray analysis showed that miR-29a-3p expression was significantly increased in the chronic phase of RE, suggesting that exosomal miR-29a-3p may be a new candidate alternative marker for chronic RE [13].

Bile acid metabolism was associated with GERD through Mendelian randomization studies, containing the bile acid metabolites glycocholic acid sulfate, glycochenodeoxycholic acid glucuronide, glycodeoxycholic acid 3-sulfate taurodeoxycholic acid 3-sulfate, deoxycholic acid 12-sulfate, and taurocholic acid sulfate [14].

Therefore, transcriptomic and metabolomic analysis were performed to investigate the mechanisms underlying quercetin’s ameliorative effects on RE.

## Materials and methods

### Materials

Quercetin (NO: B20527, purity ≥98%) was purchased from Yuanye Bio-Technology Co., Ltd. (Shanghai, China), and Omeprazole (NO: H20093291) was purchased from Renhe (Hebei, China). ELISA kits for the detection of murine IL-6 (SEA079Ra) and IL-1*β* (SEA563Ra) were provided by Cloud-Clone Corp. (Wuhan, China).

### Animals

Male Wistar rats 24, aged 6–8 weeks and weighing between 240–260 g, were obtained from Beijing SPF Biotechnology Co., Ltd, with an experimental animal license number SYXK (Jin) 2019–0002. The rats were kept in a controlled environment at a constant temperature of 25 °C, with a 12-hour light/12-hour dark cycle and 40–60% humidity range. They were provided with standard feed and sterile water ad libitum and acclimatized for seven days before the experiment. Ethical approval was obtained from the Institute of Radiation Medicine, Chinese Academy of Medical Sciences, and the number of this animal study is IRM-DWLL-2021135.

### Model establishment and drug treatments

The rats were acclimatized for 7 days and then randomly assigned numbers using a random number table, dividing them into four groups: control, model, Omeprazole, and quercetin groups. The control group underwent “laparotomy and suture surgery,” while the remaining groups underwent “cardiomyotomy plus external pyloric ligation” to establish a rat model of RE based on our previous study [15].

Briefly, the rats were anesthetized with 1.5% isoflurane, and isoflurane exposure was gradually reduced and sustained at 0.5% after loss of the righting reflex. After sterilization and skin preparation, the rats were kept in the supine position and fixed on a sterile operating table with the abdominal cavity completely exposed. To avoid bleeding during the surgery, the left gastric artery was sewn with a fine needle. A 25 mm incision was made along the median abdominal line. The muscle was cut longitudinally at the esophageal junction by 1 cm, fully exposing the mucosa. A metal rod was placed longitudinally outside of the pylorus of the stomach. A 4 mm diameter metal rod was attached to the pylorus and removed. The rats were fed 5% glucose and sodium chloride solution. After 24 h of fasting, a 15 g/day diet was administered for 3d and 30 g/day for 14 d. Feeding was then gradually resumed and changed to a normal water diet (food was sterilized by UV and water by autoclaving). Surgical sutures were disinfected daily with iodophor postoperatively to prevent incisional infection. The food, water, drinking bottles and metabolic cages were changed daily.

After 7 days, Omeprazole (30 mg/kg) and quercetin (100 mg/kg) were administered according to the doses reported in the literature [16,17]. Physiological saline dissolved quercetin and Omeprazole. The control and model groups received 2.5 mL physiological saline by gavage. The drugs were administered twice a day for 14 consecutive days.

The rats were fasted for 24 h after the last dose of the drug but were allowed to drink. Following moderate isoflurane gas anesthesia, abdominal aortic blood was collected, and final cervical dislocation euthanasia was performed. The lower part of the esophagus was removed from the cardia to the pharynx. The esophagus was incised lengthwise. The lower 2 cm of the esophagus was divided into two parts, washed with pre-cooled sterile saline, quenched in liquid nitrogen, frozen at ultra-low temperature for more than 1 h, and then transferred to a -80 °C refrigerator for spare parts. The remaining parts of the esophagus were fixed with paraformaldehyde.

### Observation of esophageal mucosa

The rat esophagus was rinsed with pre-cooled sterile saline, and the esophageal mucosa was observed macroscopically and scored according to the “Diagnosis and Treatment Guidelines for Reflux Esophagitis” issued by the Chinese Society of Digestive Endoscopy, Chinese Medical Association in 2003 (Table 1).

**Table 1:** Scoring criteria for esophageal mucosa under macroscopic observation.

Grade	Macroscopic observation	Integral
0	No significant change	0
Ia	The mucosa is red and has erosion (less than 2 sites)	1
Ⅰb	Mucosal ulceration and erosion (≥2 sites)	1.5
II	Mucosal edema with nodular changes, with fusion of less than 75%	2
III	“Bark-like” or “papillary” hyperplasia with fusion greater than 75%	3

### HE

Esophageal tissue was fixed in formaldehyde fixative solution for 48 h, dehydrated, embedded, sectioned, and stained with Hematoxylin and Eosin (HE). Pathological changes in the esophageal mucosa were observed under an optical microscope.

The rats’ esophagus was cut longitudinally, the damaged part was exposed, and the overall pathological grade of the esophagus was calculated using the diagnostic criteria for RE issued by the Chinese Medical Association Digestive Endoscopy Society in 2003 (Table 2).

**Table 2:** Scoring criteria for esophageal mucosa pathological grading.

Mucosal pathological alterations	Pathological grading		
	mild (1 point)	moderate (2 points)	severe (3 points)
Squamous epithelial hyperplasia	Yes	Yes	Yes
Extension of papillae in the lamina propria of the mucosa	Yes	Yes	Yes
Inflammatory cell infiltration	Yes	Yes	Yes
Erosion	No	Yes	No
Ulceration	No	No	No
Barrett’s esophagus changes	No	No	Yes/No

### Elisa

Whole blood without anticoagulant was collected *in vitro* and stored at room temperature for 1 h. Following coagulation and precipitation, approximately 1,500 *g* of serum was centrifuged at 4 °C for 10 min, and the clear serum was separated for analysis. The levels of IL-1*β* and IL-6 in the serum were determined using ELISA kits according to the manufacturer’s instructions.

### RNA-seq and data analysis

Esophageal RNA was extracted using TRIzol^®^ reagent (Qiagen, Germany) according to the manufacturer’s instructions. RNA quality was then determined using a 5300 Bioanalyser (Agilent) and quantified using ND-2000 (NanoDrop Technologies). Only high-quality RNA samples (OD260/280 = 1.8 ~ 2.2, OD260/230 ≥ 2.0, RNA quality number (RQN)≥6.5, 28S:18S≥1.0, > 1μg) were used for sequencing library construction.

RNA was purified, reverse-transcribed, library-constructed, and sequenced at Shanghai Majorbio Bio-pharm Biotechnology Co., Ltd. The RNA-seq transcript library from rat esophageal tissue was prepared using 1 μg of total RNA according to Illumina^®^ Stranded mRNA Prep, Ligation (San Diego, CA). Messenger RNA was isolated using the poly(A) selection method with oligo(dT) beads and subjected to fragmentation in a fragmentation buffer. Second, cDNA was synthesized using a SuperScript double-strand cDNA Synthesis Kit (Invitrogen, CA) with randomized hexameric primers. The synthesized cDNA was subjected to end-repair, phosphorylation, and adapter ligation according to the library construction protocol. The library was size-selected for cDNA targets of 300 bp on 2% Low Range Ultra Agarose, and PCR amplification was performed using Phusion DNA Polymerase (NEB) for 15 PCR cycles. Library sequencing was performed on the NovaSeq X Plus platform (PE150) using the NovaSeq Reagent Kit after quantification with Qubit4.0.

Raw paired-end reads were trimmed by fastp with default parameters, and quality control was performed. The HISAT2 software was then used to separately align the clean reads to the reference genome in orientation mode. Each sample’s mapped reads were reference assembled using StringTie. To identify differential expression genes (DEGs) between two different samples, the expression level of each transcript was quantified using the transcripts per million reads (TPM) method. RSEM was used to quantify gene abundance. Differential expression analysis was performed using DESeq2. DEGs with |log2FC|≧ 1 and FDR < 0.05 were considered significant DEGs. Functional enrichment analysis including Gene Ontology (GO) and Kyoto Encyclopedia of Genes and Genomes (KEGG) was also performed to identify which DEGs were significantly enriched for GO terms and pathways with Bonferroni corrected *P* < 0.05 compared to the total transcriptome background. Goatools and Python scipy software were used for GO functional enrichment and KEGG pathway analysis, respectively. All alternative splicing events occurring in our sample were identified using the rMATSl7 program. Exons were included and excluded, and alternative 5’, 3’, and intronic retention events were detected.

The raw esophageal transcriptome sequencing data involved in the manuscript has been uploaded to NCBI (https://www.ncbi.nlm.nih.gov/) under SRA number PRJNA1203977.

### Metabolite extraction and UHPLC-MS/MS analysis

50 mg esophageal tissue was added to a 2-mL centrifuge tube, and a 6-mm-diameter grinding bead was added. For the extraction of metabolites, 400 μL of extraction solution (methanol: water = 4:1 (v:v)) containing 0.02 mg/mL of internal standard (L-2-chlorophenylalanine) was used. Samples were ground using the Wonbio-96c frozen tissue grinder (Shanghai Wanbo Biotechnology Co., LTD) for 6 minutes (-10 °C, 50 Hz), followed by low-temperature ultrasound extraction for 30 minutes (5 °C, 40 kHz). Samples were left at -20 °C for 30 minutes, centrifuged for 15 minutes (4 °C, 13,000g), and the supernatant was added to the injection vial for LC-MS/MS analysis.

For system conditioning and quality control purposes, equal volumes of all specimens were mixed to form one pool for quality control (QC). The QC samples were processed and tested in the same way as the analytical samples. They represented the full set of samples that would be injected at regular intervals (every 5–15 samples) for the stability of analysis monitoring.

LC-MS/MS was performed on a Thermo UHPLC-Q Exactive HF-X system equipped with an ACQUITY HSS T3 column (100 mm × 2.1 mm i.d., 1.8 μm; Waters, USA) at Majorbio Bio-Pharm Technology Co. Ltd. (Shanghai, China). The mobile phases were 0.1% formic acid in water: acetonitrile (95:5, v/v) (solvent A) and 0.1% formic acid in acetonitrile: isopropanol: water (47.5:47.5, v/v) (solvent B), with a flow rate of 0.40 mL/min and a column temperature of 40°C. Mass spectrometry data were collected using a Thermo UHPLC-Q Exactive HF-X mass spectrometer with an electrospray ionization source. Optimal conditions were set at 425°C source temperature; 50 arb sheath gas; 13arb aux gas; ion-spray voltage floating at -3500V in negative mode and 3500V in positive mode; 20–40-60V rolling collision energy for MS/MS. The full MS resolution was 60,000, and the MS/MS resolution was 75,000. Data acquisition was in DDA mode, and detection was 70–1050 m/z.

Data was analyzed using majorbio cloud (cloud.majorbio.com) [18]. LC/MS raw data was treated using Progenesis QI software (Waters Corporation, Milford, USA), and a three-dimensional CSV matrix was exported. At the same time, the metabolites were identified by searching databases, and the main databases were the Human Metabolome Database (HMDB) (http://www.hmdb.ca/), Metlin (https://metlin.scripps.edu/), and Majorbio Database. The database search data matrix was uploaded to the Majorbio cloud platform (https://cloud.majorbio.com) for analysis. The data matrix was pre-processed as follows: At least 80% of the metabolic features were retained. After filtering, for samples with low metabolite levels, the minimum was estimated, and each metabolic signature was normalized to the sum. To reduce errors caused by sample preparation and instrument instability, the sum normalization method was used to normalize the response intensities of peaks in the mass spectrometry. The QC samples with >30% RSD were excluded and log10-transformed to get the final data matrix for analysis.

The R package “ropls” (version 1.6.2) was then used to perform xorthogonal least partial squares discriminant analysis orthogonal Partial Least Squares Discriminant Analysis (OPLS-DA) and 7-cycle interactive validation to assess the model’s stability. Metabolites with VIP > 1, *P* < 0.05 were determined as significantly different metabolites by OPLS-DA and student’s t-test. Differential metabolites between two groups were mapped into their biochemical pathways by metabolic enrichment and pathway analysis based on the KEGG database (http://www. genome.jp/kegg/).

Integrated transcriptomic and metabolomic analyses have been performed using iPath 3.0 to profile significantly altered metabolic pathways. Metabolomics data are fully available in the EMBL-EBI MetaboLights repository under accession number MTBLS12138 (https://www.ebi.ac.uk/metabolights/MTBLS12138).

### Statistical analysis

The experimental data were expressed as mean ± standard deviation, and the data were analyzed by the GraphPad Prism 9.0 software. If normal distribution and homogeneity of variance were fulfilled, one-way ANOVA was used for comparison among control, model, quercetin groups. Otherwise, Kruskal-Wallis test was used for comparison among control, model, quercetin groups. *P* < 0.05 was considered statistically significant.

## Results

### Quercetin attenuated esophageal injury in RE rats

**Fig 1:**
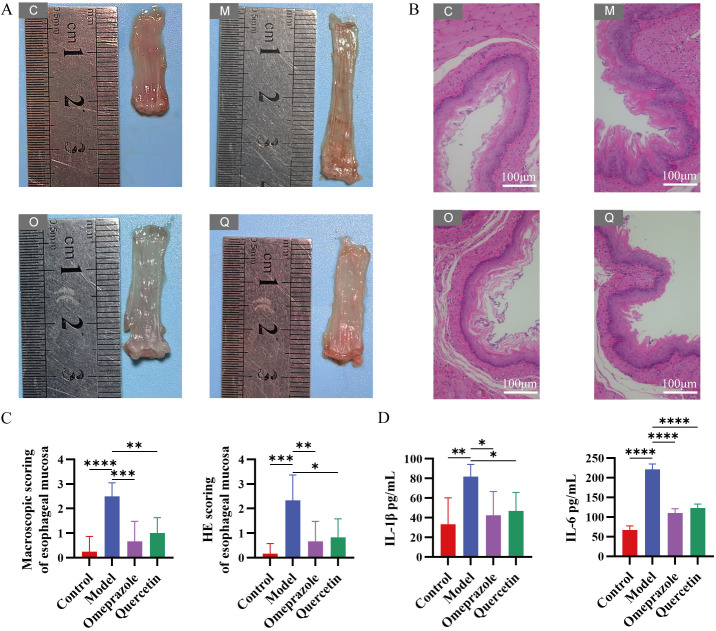
Quercetin attenuated esophageal injury in RE rats. **(A)** Images showed the esophagus after treatment with the control group, model group, Omeprazole group, and quercetin group. **(B)** H&E stained images of the sectioned esophagus tissues from rats with different groups. **(C)** Macroscopic and HE scores of the esophagus. **(D)** The expression of IL-1*β* and IL-6 in rats was measured by Elisa. C-Control, M-Model, O-Omeprazole, Q-Quercetin. *n* = 6, ^*^P < 0.05, ^**^P < 0.01, ^***^P < 0.001, ^****^P < 0.0001.

Rats in the control group showed no evidence of RE lesions on gross examination or HE staining images of the esophagus. In contrast, under the naked eye, the esophageal mucosal surface of the RE model group was uneven, with congestion and erosion (Fig 1A). The esophageal mucosal epithelium of the model group showed squamous epithelial hyperplasia, many inflammatory cell infiltrates, and elongation of the lamina propria papilla. Compared with the model group, the esophageal mucosa of the quercetin group was improved in terms of inflammatory cell infiltration, squamous epithelial thickening, and lamina propria papilla elongation, and the Omeprazole group had similar results (Fig 1B-C). In comparison with the RE model group, quercetin significantly reduced the expression of the inflammatory factors IL-1β and IL-6, and similar results were found in the Omeprazole group (Fig 1D).

### Effect of quercetin on esophageal transcriptomics

**Fig 2:**
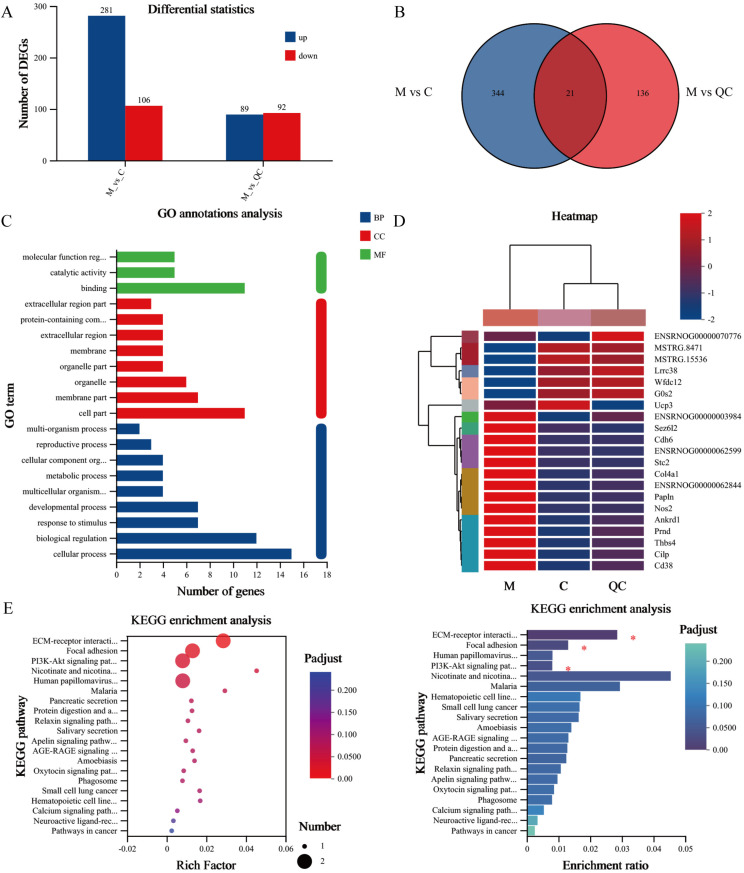
Effect of quercetin on transcriptomics in rats with RE. **(A)** Numbers of upregulated and downregulated DEGs in different groups. **(B)** Venn diagram of DEGs in different groups. **(C)** GO functional annotation analysis of DEGs. **(D)** Heatmap of the DEGs. Red indicates highly expressed genes and blue indicates less expressed genes. **(E)** Enrichment analysis of the DEGs. The degree of enrichment increases with a higher rich factor, with the color gradient of the bar representing the significance of this enrichment. *n* = 5, ^*^P < 0.05.

Esophageal samples from control, model, and quercetin groups were analyzed by RNA-Seq. Differential expression analysis revealed 387 differential expression genes (DEGs) in the model group compared to the control, with 281 genes upregulated and 106 genes downregulated. Following quercetin treatment, 181 DEGs were detected, of which 89 genes were downregulated and 92 genes were upregulated (Fig 2A). The 21 overlapping DEGs in the control, model and quercetin groups were represented by Venn diagrams (Fig 2B). Gene Ontology (GO) functional annotation and heat mapping of the 21 DEGs indicated quercetin may be involved in biological regulation, metabolic process, and molecular function regulation (Fig 2C). Interestingly, genes that were upregulated or downregulated in the model group exhibit corresponding downregulation or upregulation in the quercetin group, exemplified by cartilage intermediate layer protein (*Cilp*) and nitric oxide synthase 2 (*Nos2*) (Fig 2D). Kyoto Encyclopedia of Genes and Genomes (KEGG) pathway analysis of the DEGs suggested that quercetin may function by regulating the phosphatidylinositol 3kinase (PI3K)/ protein kinase B (AKT) signaling pathway and nicotinic acid and nicotinamide metabolism (Fig 2E).

### Metabolomics of quercetin in rats with RE

**Fig 3:**
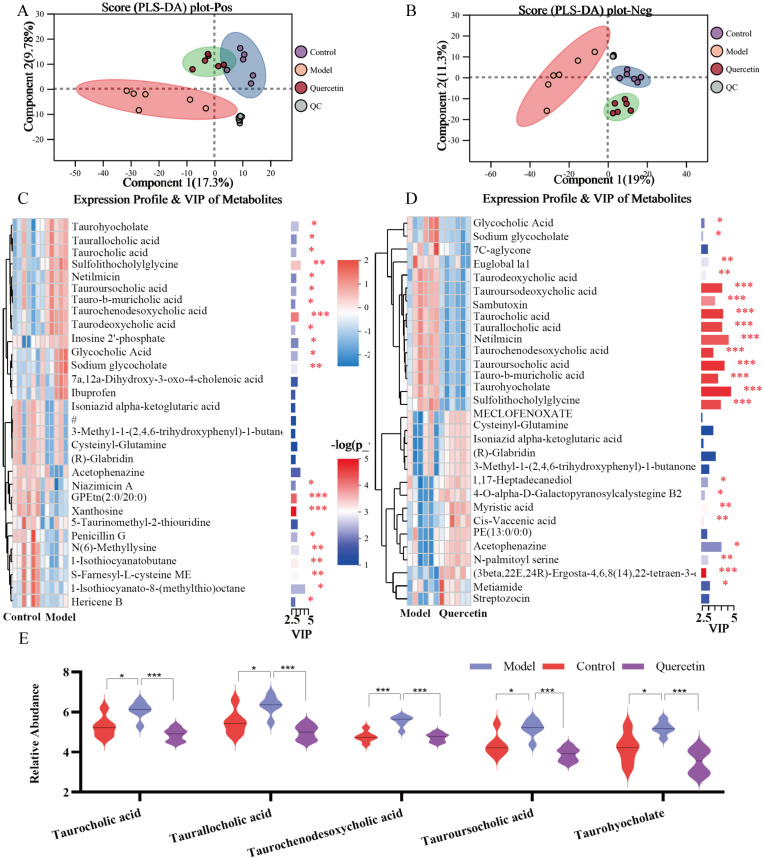
Metabolic effects of quercetin in rats with RE. **(A-B)** PLS-DA plot of positive and negative symbols. **(C-D)** Heat map of VIP analysis of metabolites. Each row represents a metabolite and the color indicates its relative expression level. Redder shades indicate signify higher metabolite content. VIP values greater than or equal to 1, with higher values indicating greater metabolic differences. **(E)** The relative expression levels of metabolites with significant differences. *n* = 6, ^*^P < 0.05, ^**^P < 0.01, ^***^P < 0.001.

**Fig 4:**
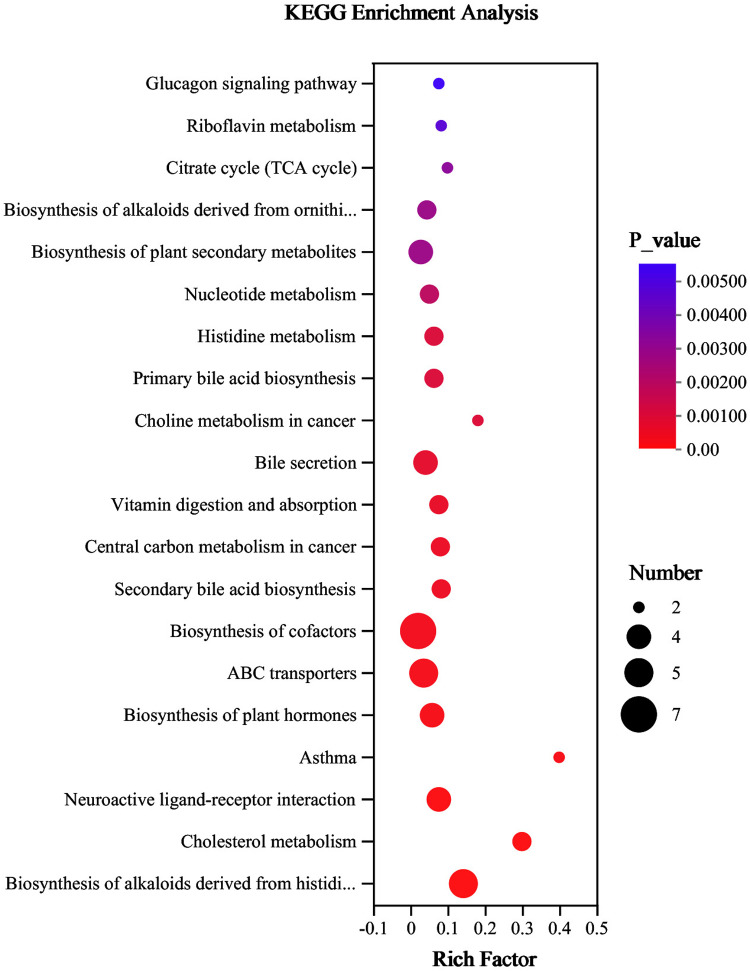
KEGG enrichment analysis of differential metabolites between model and quercetin groups.

Further metabolomic analysis of esophageal tissues was performed because the improvement in RE by quercetin was related to metabolic processes based on transcriptome analysis. Metabolites from rats in the model group were separated from the control and quercetin groups, whereas metabolites from the quercetin and control groups were closer together (Fig 3A-B). Thus, quercetin modified the metabolic changes associated with RE injury. In total, 204 differential metabolites were found after modeling, 137 metabolites were found after quercetin treatment, and the intersection yielded 88 differential metabolites (S1 Fig). According to the classification of the Human Metabolome Database (HMDB) database, 88 differential metabolites were found, including glycerophosphocholines, bile acids, carbohydrates, and carbohydrate conjugates (S2 Fig). Heat map analysis of the differential metabolites revealed that quercetin may inhibit inflammation by decreasing glycocholic acid, taurocholic acid and sulfolithocholylglycine, while increasing isoniazid α-ketoglutaric acid and myristic acid (Fig 3C-D). The top five metabolites with significant differences in relative abundance were taurocholic acid, taurallocholic acid, taurochenodesoxycholic acid, tauroursocholic acid, and taurohyocholate (Fig 3E). In addition, KEGG enrichment analysis showed that differential metabolites between the model and quercetin groups were related to cholesterol metabolism, bile acid secretion and biosynthesis, and histidine metabolism, consistent with the VIP metabolism results (Fig 4). Bile acids, the main constituents of bile, are divided into free and conjugated forms, with the free bile acids being able to form taurine conjugates such as taurocholic acid when combined with glycine or taurine [19]. In conclusion, the mechanism of quercetin inhibition of RE may be related to bile acid metabolism.

### Integrated analysis of transcriptomics and metabolomics in bile metabolism

**Fig 5:**
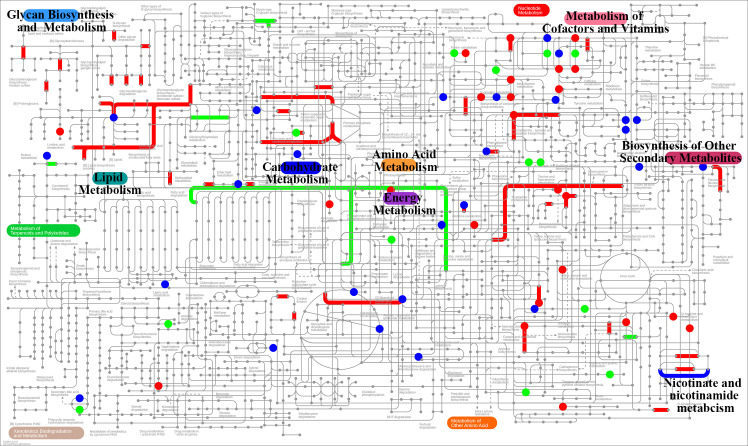
Integrated transcriptomic and metabolomic analysis in iPath 3.0 to visualize the KEGG general metabolic pathway maps. Dots and lines represented differential metabolites and DEGs, respectively. Red, model vs. control; green, quercetin vs. model; blue, shared metabolites and DEGs.

The metabolic pathways altered by the quercetin intervention were further explored by integrating the differential metabolites in iPath 3.0 for in-depth analysis (Fig 5), and the alterations in metabolic pathways were mainly enriched in nicotinic acid and nicotinamide metabolism, similar to the transcriptome analysis. Niacin and nicotinamide are the two forms of Vitamin B3. Niacin, also known as nicotinic acid, and nicotinamide, also known as niacinamide, are amide compounds of niacin. Nicotinamide is a chemical component of coenzyme I (NAD^+^) and coenzyme II (NADP^+^), the reduced forms of which are nicotinamide adenine dinucleotide (NADH) and nicotinamide adenine dinucleotide phosphate (NADPH), respectively, and most cellular metabolic reactions cannot occur without NAD^+^ and NADP^+^.

Bile acids can activate the epidermal growth factor receptor (EGFR), and activation of EGFR also leads to upregulation of cyclooxygenase (COX) and lipoxygenase (LOX), which promotes the production of reactive oxygen species (ROS) and causes inflammation. Bile acids can disrupt the plasma membrane and release arachidonic acid into the cytoplasm, where it is rapidly used as a substrate for the biosynthesis of COX and LOX enzymes. NADPH oxidase can be stimulated by bile acids, further increasing the production of ROS, which causes inflammation and leads to the upregulation of the expression of the pro-inflammatory cytokine IL -1*β* [19].

**Fig 6:**
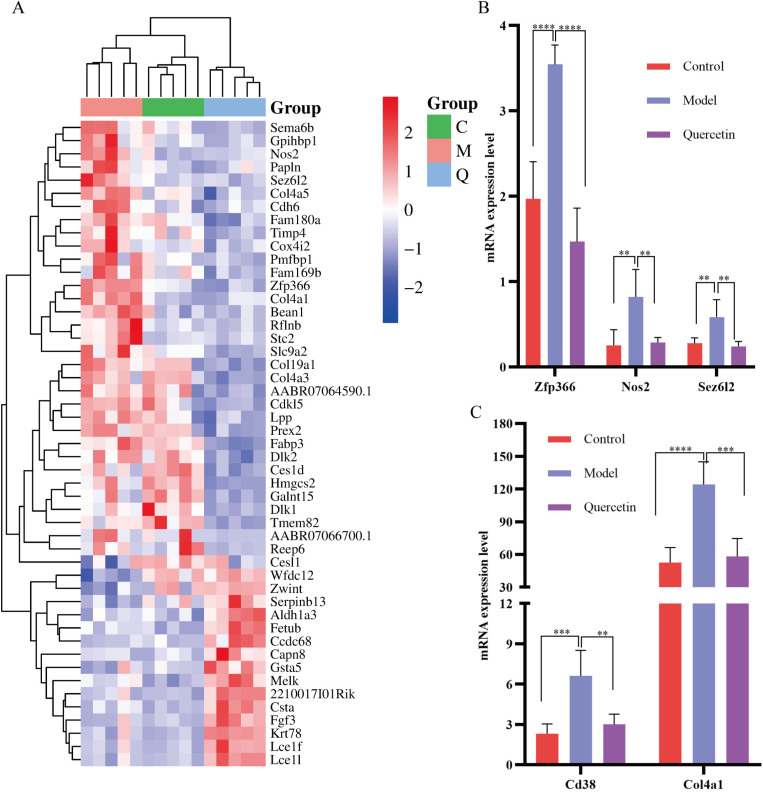
Integrated transcriptomic and metabolomic analysis of bile acid metabolism in RE rats. **(A)** The heatmap of genes related to bile acid metabolism. Red indicated highly expressed genes and blue indicated less expressed genes. **(B-C)** Histogram of mRNA expression of genes with significant differences in correlation with bile acid metabolism. *n* = 5, ^**^P < 0.01, ^***^P < 0.001, ^****^P < 0.0001.

Based on the transcriptomic and metabolomic analysis, it was speculated that quercetin ameliorates esophagitis by regulating bile acid metabolism. The correlation of genes and metabolites involved in the enrichment pathway was comprehensively analyzed by Pearson arithmetic, with coefficients closer to 1 indicating that the genes were more correlated with the metabolites. The DEGs of the taurine enrichment pathway, which had a positive correlation, were further analyzed and represented by using a heat map (Fig 6A). Quercetin significantly reduced the expression of *Cd38*, zinc finger protein 366 (*Zfp366*), collagen type iv alpha 1 chain (*Col4a1*), *Sez6l2,* and *Nos2* mRNA in the model group (Fig 6B-C). However, further experiments are needed to prove this.

## Discussion

GERD represents a common gastrointestinal disorder resulting from abnormal reflux of gastric contents, possibly due to excessive dietary fat intake or maladaptive eating patterns [20]. One common manifestation of GERD is RE, which causes inflammation of the esophagus. Symptoms include heartburn, chronic cough, throat irritation, and even asthma. In this study, a rat model of RE was established, and it was found that quercetin could alleviate the symptoms of RE in rats, including improved food intake and activity status. A reduction in esophageal tissue lesions was also observed, which is consistent with previous research findings [11]. The cytokine IL-6 has been implicated in the pathophysiology of GERD and acts as an important mediator in controlling inflammatory responses. Similarly, in both animal models of esophageal inflammation and patients with GERD, elevated levels of IL-1*β* had been observed in the esophageal mucosa and muscle layers [21]. Some studies have reported that mucosal damage in gastro-esophageal reflux isn’t a direct causative effect of gastric acid, but rather promotes the secretion of inflammatory cytokines that cause epithelial cell damage [22]. Quercetin, therefore reduced the release of pro-inflammatory factors in reflux, with some therapeutic significance.

According to JAMA, RE is caused not only by acid stimulation but also by immune factors [23]. Nitric oxide (NO) is an inflammatory mediator. NO produced by iNOS (also known as Nos2) is pro-inflammatory [24]. Under conditions of ischemia and hypoxia, iNOS is activated in macrophages and leukocytes, producing significant amounts of NO, which can lead to cellular damage and mucosal inflammatory responses [25]. Through the comprehensive transcriptomic and metabolomic analysis, we identified *Nos2* as a differentially expressed gene that was significantly upregulated in the model group, whereas its expression was downregulated in both the control and quercetin-treated groups.

The transcriptome and metabolome studies of the protective effect of quercetin in RE provided strong technical support. KEGG analysis of the DEGs revealed that the major KEGG pathways enriched for quercetin included the PI3K/AKT pathway, Nicotinate metabolism, relaxin pathway, calcium pathway and AGE/RAGE pathway. The PI3K/AKT pathway has been reported to be closely associated with RE development, and atractylenolide Ⅲ attenuated RE development by inhibiting the PI3K/AKT/NF-κB/iNOS pathway [26,27]. Niacin and nicotinamide metabolism are also involved in inflammation, and bile acids stimulate NADPH oxidase production, which in turn stimulates ROS and NF-κB, leading to inflammation [28,29].

Bile acids are the main components of bile and help in the digestion and absorption of fats. However, when bile acids reflux into the esophagus, they damage the lining of the esophagus, causing RE. The average concentration of bile salts in the stomach of normal subjects ranges from 50 ~ 500 μmol/L to 9 mmol/L, and the presence of bile salts in the stomach suggests a similar concentration of bile salts in the esophageal reflux [30].

Bile acids are essential physiological regulators of nutrient absorption from the gut and the excretion of lipids, toxic metabolites, and exogenous agents. Bile acids are also signaling molecules and metabolic regulators, activating nuclear and G protein-coupled receptor signaling, regulating hepatic lipid, glucose, and energy homeostasis, and maintaining metabolic homeostasis. Toxic bile acids cause inflammation and cell death and can lead to liver disease, dyslipidemia, fatty liver, cardiovascular disease, and diabetes [31]. The link between bile and esophagitis was first suggested in 1957 by Cross and Wangenstein using cat and dog models. They transferred bile directly from the duodenum to the esophagus, causing severe esophageal damage in the absence of gastric acid [30]. Researchers have observed that bile acid reflux occurs within a dynamic pH spectrum, with most of these episodes occurring simultaneously with acid reflux, a phenomenon known as mixed reflux (acid and bile acid). This mixed reflux pattern is a significant contributor to severe esophageal mucosal injury. It was found that in children with RE, bile acid reflux alone accounted for 22.7% of cases, and reflux due to a mixture of bile and gastric acid accounted for 36.4% [32].

Bile acid metabolism plays an important role in the development and progression of RE. Metabolism produces certain bile acids such as deoxycholic acid, a bile acid metabolite of intestinal bacteria which is highly toxic and can damage the esophageal mucosa by producing ROS, activating the NF-κB pathway and reducing the adhesion of esophageal epithelial cells [22,33]. Several studies have reported that conjugated bile acids, including taurocholate and glycocholic acid, are the major bile acids in the esophageal refluxate of patients with esophagitis [34]. The main constituents of the refluxate include primary bile acids, specifically taurocholic acid, glycocholic acid, and cholic acid, which play a key role in the pathogenesis of esophageal damage [35]. Our results were consistent with previous research, as the RE model group showed a significant increase in taurocholic acid levels, whereas the quercetin-treated group showed a decrease.

Metabolomic analysis showed that the differential metabolites were mainly glycerophosphorylcholine, bile acids, and amino acids. Treatment of RE rats with quercetin significantly improved RE-induced metabolic disturbances of alkaloids, glucagon, riboflavin, bile acids, and cholesterol. Further analysis revealed quercetin’s amelioration of RE was more associated with bile acid metabolism, such as taurocholic acid and taurine. Interestingly, it was also found to be associated with niacin and nicotinamide metabolism, and NADPH (of which nicotinamide is a component) has been reported to be involved in inflammation with bile acids [28].

The *Cd38* gene encodes a protein that is normally localized to the cell surface, and Cd38 catalyzes the synthesis of nicotinamide using NAD^+^ as a substrate. *Cd38* has been reported to be strongly induced in infection and subsequent inflammation [36], and *Cd38* expression is transcriptionally controlled by TNFα/NF-κB stimulation [37]. *Cd38* is highly expressed in esophageal inflammatory tissue [38], and therefore, is clearly associated with esophagitis. Sez6l2 is a brain-specific receptor-like protein that is mainly expressed in the cytosol and dendrites of neurons and is required for synapse maturation [39]. Previous studies have shown that Sez6l2 is highly expressed in carcinoma cells of the lung, cholangiocarcinoma, colon, and breast, and serves as a negative prognostic marker for a variety of tumors [40–43]. Inflammation is closely associated with tumors, and Sez6l2 may also play an important role in reflux esophagitis [44]. Perhaps, above genes, could be characteristic genes and new targets for intervention in reflux esophagitis, and quercetin may play a role in regulating bile acid metabolism and suppressing reflux esophagitis by targeting them. Of course, more research is needed to confirm this.

Quercetin’s value in medicine and nutrition is well-known, with potential applications in many fields. However, note that, despite its health benefits, its dosage, stability and interaction with other components must be considered in practical applications. Quercetin has low solubility and bioavailability, and poor stability. Higher doses are needed to achieve the desired effect [45]. Quercetin may promote the proliferation of estrogen-related tumor cells and adverse effects on prerenal renal function. Concerning the occurrence of adverse effects in human intervention studies, studies have investigated high doses of quercetin, up to 1000 mg per day, either as a single compound or in combination with vitamin C, for a maximum duration of 12 weeks, with a very low incidence of adverse effects [46]. Further studies are needed to elucidate its mechanism of action, optimize its bioavailability and assess its long-term safety.

In patients with non-acid reflux, biliary diversion surgery may be considered, as well as the search for drugs to modulate bile acid metabolism, decreasing toxic bile acid metabolites and increasing beneficial bile acid metabolites, thus facilitating the improvement of RE.

The study has several limitations. The first and most fundamental is that further mechanistic studies are needed. Multiple concentration gradients of quercetin were used in order to explore whether it alleviated esophageal injury in a dose-dependent manner. Furthermore, the effect of quercetin on RE was observed at more time points. Animal studies of bile acid esophagitis and further trials in RE patients are needed to address these limitations. We will address these in our future study.

## Conclusion

Quercetin may act as a suppressor of the RE by regulating the metabolism of bile acids.

## Supporting Information

S1 Fig Venn diagram differential metabolites between groups. Subsequent to the establishment of the model, a total of 204 distinct metabolites were identified. Similarly, after the administration of quercetin, 137 unique metabolites were observed. Upon intersecting these two datasets, 88 metabolites were found to exhibit differential changes in both scenarios.
(TIF)

S2 Fig HMDB classification of differential metabolites. Differential metabolites include glycerophosphocholines, bile acids, carbohydrates and carbohydrate conjugates, glycerophosphoethanolamines, and amino acids.
(TIF)
