# Supplementary figures and images for: Integrated transcriptomic and metabolomic analyses reveal the mechanism by which quercetin inhibits reflux esophagitis in rats
Source: PLoS One. 2025 May 6;20(5):e0321959. doi: 10.1371/journal.pone.0321959 (PMC12054900; doi:10.1371/journal.pone.0321959)

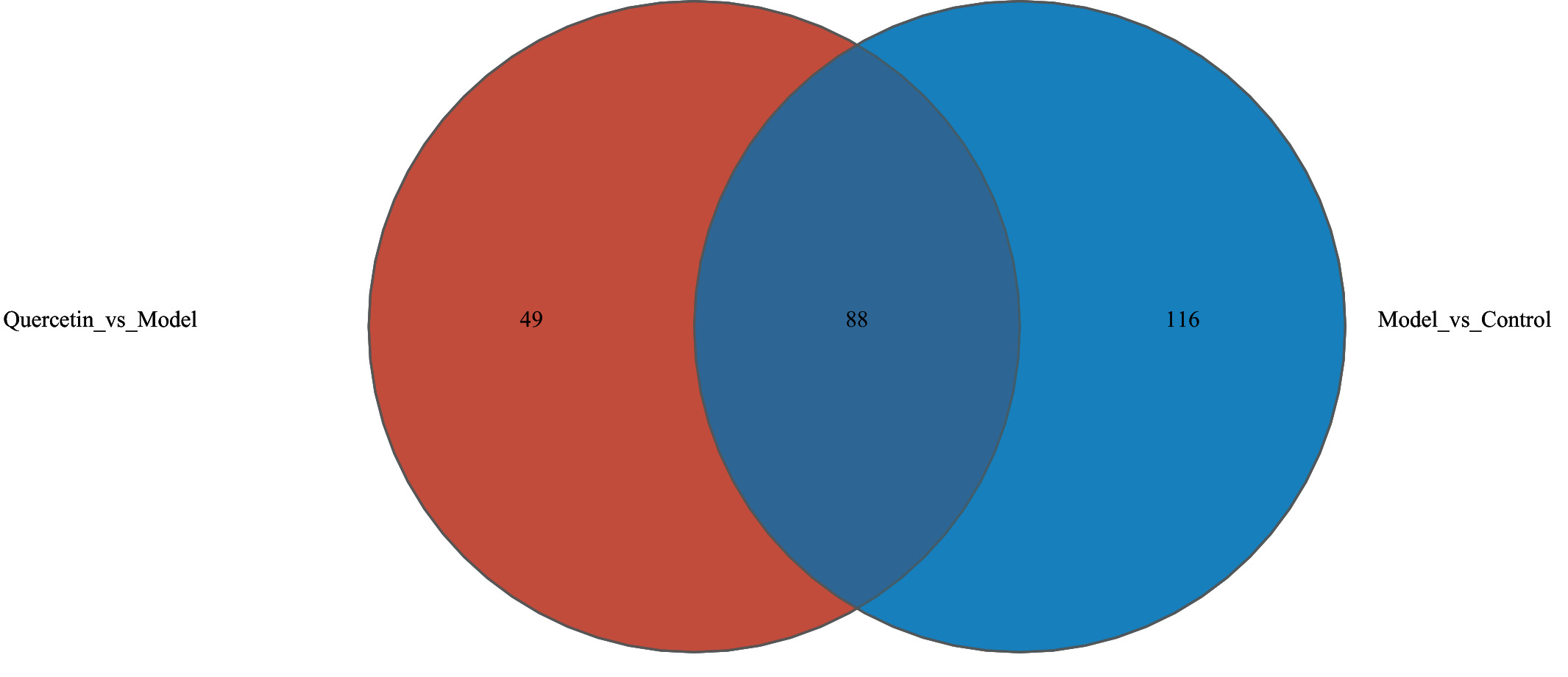

Supplement: S1 Fig — Subsequent to the establishment of the model, a total of 204 distinct metabolites were identified. Similarly, after the administration of quercetin, 137 unique metabolites were observed. Upon intersecting these two datasets, 88 metabolites were found to exhibit differential changes in both scenarios. (TIF) [file pone.0321959.s001.tif]

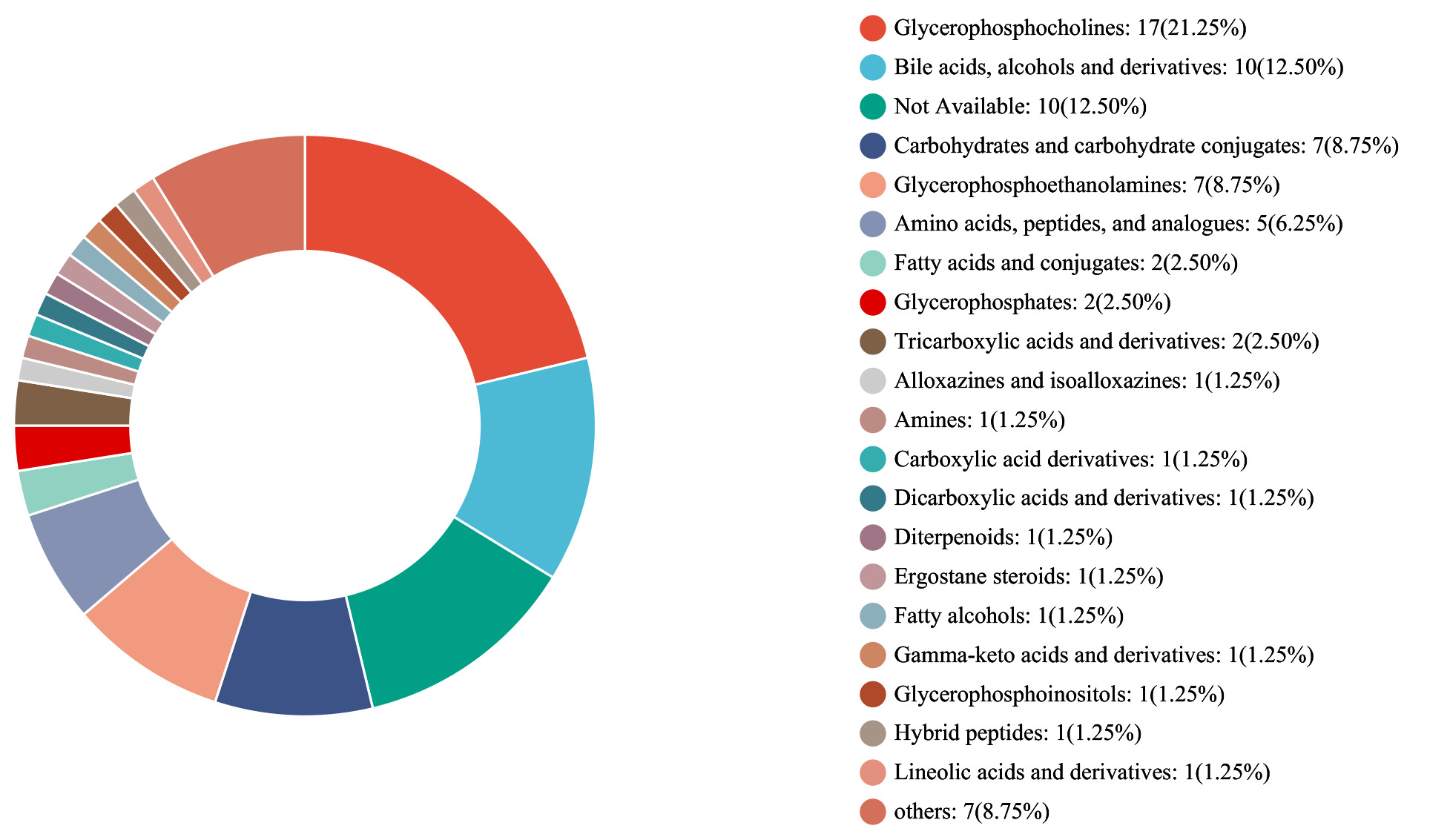

Supplement: S2 Fig — Differential metabolites include glycerophosphocholines, bile acids, carbohydrates and carbohydrate conjugates, glycerophosphoethanolamines, and amino acids. (TIF) [file pone.0321959.s002.tif]
